# Visceral fat area is more strongly associated with arterial stiffness than abdominal subcutaneous fat area in Chinese patients with type 2 diabetes

**DOI:** 10.1186/s13098-024-01356-2

**Published:** 2024-06-05

**Authors:** Jing Mao, Shenglian Gan, Shijun Gong, Quan Zhou, Fang Yu, Haifeng Zhou, Huilin Lu, Qian Li, Zhiming Deng

**Affiliations:** 1 Department of Science and Education, Changde Hospital, Xiangya School of Medicine, Central South University, Changde, China; 2 Department of Endocrinology, Changde Hospital, Xiangya School of Medicine, Central South University, Changde, China; 3 Department of Ultrasound, Changde Hospital, Xiangya School of Medicine, Central South University, Changde, China; 4 Department of Pulmonary and Critical Care Medicine, Changde Hospital, Xiangya School of Medicine, Central South University, Changde, China

**Keywords:** Visceral fat area, Abdominal subcutaneous fat area, Type 2 diabetes, Arterial stiffness, Insulin resistance

## Abstract

**Background:**

Few studies have compared the correlation between visceral fat area (VFA) and abdominal subcutaneous fat area (SFA) with arterial stiffness (AS) in patients with type 2 diabetes (T2D). In addition, there is currently controversy regarding the correlation between VFA and SFA with AS. We aimed to investigate the relationship between VFA and SFA with AS in patients with T2D.

**Methods:**

In this cross-sectional study, 1475 Chinese T2D patients with an average age of 52.32 ± 10.96 years were included. VFA and SFA were determined by a dual bioelectrical impedance analyzer, and AS was determined by measurement of brachial-ankle pulse wave conduction velocity (baPWV). Atherosclerosis was deemed present in study participants with baPWV values higher than 75th percentile (1781 cm/s). Independent correlations of logVFA and logSFA with AS were assessed using multiple linear regression and multivariate logistic regression.

**Results:**

The baPWV was linked with VFA, waist circumference, and women’s SFA in a general linear correlation study (*P* < 0.05), but not with body mass index (*P* = 0.3783) or men’s SFA (*P* = 0.1899). In both men and women, VFA and SFA were positively correlated with AS, according to the generalized additive model (GAM). After fully adjusting for confounders, multiple linear regression analyses showed that for every 1-unit increase in logVFA, the beta coefficient of baPWV increased by 63.1 cm/s (95% CI: 18.4, 107.8) (*P* < 0.05). logSFA did not correlate significantly with baPWV (*P* = 0.125). In the multiple logistic regression analysis, the odds ratio (OR) of elevated baPWV was 1.8 (95% CI: 1.1, 3.1) (*P* = 0.019) per 1-unit increase in logVFA. logSFA did not correlate significantly with AS (*P* = 0.091). In the subgroup analysis, the correlation between logVFA and baPWV did not interact across subgroups (P-interaction > 0.05).

**Conclusions:**

Compared with SFA, VFA had a stronger independent positive correlation with AS in Chinese T2D patients. Patients with T2D should pay more attention to monitoring VFA and lowering it to minimize cardiovascular events.

## Introduction

Currently, cardiovascular disease (CVD) remains the leading cause of death in patients with diabetes. Since diabetics often combine important risk factors for CVD, such as hypertension and dyslipidemia, they have a two- to four-fold increased risk of developing CVD [1]. Studies have shown that the prevalence of diabetes is increasing, reaching 10.5% of adults globally in 2021, or 536.6 million people, and this number is expected to increase by 51% by 2045 [2]. In addition, there are racial differences in genetic susceptibility to type 2 diabetes (T2D) among Chinese. Compared with Caucasians, Asian populations have a 60% increased risk of diabetes [3]. Arterial stiffness (AS) is a significant CVD event risk factor that is closely linked to the emergence and progression of diabetes complications.

Cardiovascular risk is associated with the location of abdominal fat accumulation, which is composed of two main components: the visceral fat area (VFA) and the abdominal subcutaneous fat area (SFA) [4]. The majority of earlier research, which was done on healthy populations, primarily examined the relationship between VFA and AS only and did not consider the effect of SFA on the results. The correlation between SFA and AS is currently understudied and still controversial [5–8]. In addition, the correlation of VFA and SFA with AS has been less well reported in a special population of Chinese patients with T2D, who are more prone to insulin resistance (IR), hyperinsulinemia, and abnormal accumulation of fat than healthy individuals. Although the total obesity rate of Asians is lower than that of Africans and Caucasians, Asians are more prone to VFA accumulation. A complex combination of factors, including the sedentary lifestyle of the Chinese, reduced physical activity, and specific dietary patterns (e.g., refined carbohydrates and higher saturated fats), may lead to increased AS and a higher incidence of CVD [4, 9].

Numerous studies have demonstrated that AS has a stronger correlation with abdominal obesity than with overall obesity, despite the fact that obesity is a major risk factor for CVD [10]. Adults in China with a body mass index (BMI) ≥ 28 kg/m^2^ are defined as overall obesity. Men with a waist circumference (WC) ≥ 90 cm and women with a WC ≥ 85 cm are defined as abdominal obesity [11]. Some studies have shown a stronger correlation between VFA and AS than BMI and WC [12, 13], while others have reported no significant correlation between BMI and WC and AS [5, 6, 10, 14]. Due to the inability of BMI and WC to differentiate body fat distribution, the aim of this study was to compare the correlation of VFA and SFA with AS in patients with T2D in order to identify the risk factors for CVD. This will provide a practical approach and theoretical foundation for T2D patients’ CVD prevention.

## Materials and methods

### Study design and participants

Data were collected from May 1, 2020, to January 31, 2022, for this study. Participants’ data were collected by the Metabolic Management Center (MMC) in accordance with China’s national standards at the First People’s Hospital of Changde City, Hunan Province [15]. 1665 people with diabetes, ages 18 to 80, who fulfilled the 2020 American Diabetes Association’s diagnostic criteria for the disease were included in this cross-sectional study. Diabetes mellitus is defined as fasting plasma glucose (FPG) ≥ 7.0 mmol/L, 2 h postprandial plasma glucose (PPG) ≥ 11.1 mmol/L, or glycated hemoglobin (HbA1c) ≥ 6.5% [16]. Patients with type 1 diabetes (*n* = 18), age < 18 years (*n* = 3), other types of diabetes (*n* = 5), malignancy (*n* = 10), missing baPWV data (*n* = 44), low ankle-brachial index (< 0.9) (*n* = 12), and no bioelectrical impedance analysis (BIA) measurements performed (*n* = 98) were among the exclusion criteria. A final total of 1475 patients aged 18 to 80 diagnosed with T2D were included.

### Measurements of variables

All variables were measured and registered by trained researchers using standardized questionnaires, including sex, age, smoking (never, former, or current), alcohol consumption (never, former, or current), work status, duration of diabetes mellitus, antihyperlipidemic medication, antihypertensive medication, antihyperglycemic medication, as well as menopause status. Height and weight were measured after removing shoes; a non-elastic tape measure was used to measure the WC; and after the individual had been sitting motionless for at least five minutes, the average of the two measurements of systolic blood pressure (SBP) and diastolic blood pressure (DBP) [13]. The formula for calculating BMI was weight in kilograms divided by height in square meters. Laboratory data, including total cholesterol (TC), triglycerides (TG), high-density lipoprotein cholesterol (HDL-C), low-density lipoprotein cholesterol (LDL-C), FPG, fasting C peptide (FCP), and HbA1c, were measured after participants fasted overnight for at least 12 h. PPG was obtained by taking venous blood samples 2 h after the steamed bun meal and was used to assess the post-load blood glucose.

### Measurement of VFA and SFA

VFA and SFA were measured by a trained physician using a dual BIA (HDS-2000 DUALSCAN; Omron Healthcare, Kyoto, Japan) after subjects had fasted for at least 12 h [17]. The gadget measures the surface impedance of the body and the torso using a bioelectrical impedance assembly, and it also includes an abdominal dimension measurement device. Four limb electrodes were clamped to the wrists and ankles to measure trunk impedance by current and voltage, and a belt containing the electrodes was wrapped along the umbilicus to measure surface impedance. The instrument automatically calculates the body fat content based on the time and intensity of the current passage.

### Assessment of baPWV

In this study, we used the baPWV to show whether a patient had atherosclerosis or not [18]. The baPWV measures were taken by the technician using an automatic atherosclerosis detection instrument (model HBP-8000, Omron Healthcare (China) Co., Ltd.) while the subjects were resting in the supine position for a minimum of five minutes. The device automatically calculated the distance from the humerus to the ankle based on the subject’s height. The arm band of the automatic recording instrument was wrapped 2–3 cm above the crossbar of the elbow socket, and the ankle band was wrapped 1–2 cm above the inner ankle. The machine automatically measures the time difference between the systolic pulse waveforms. The baPWV was computed by dividing the distance between the two spots by the time difference [2, 19]. We used the average of the left and right sides of the baPWV for analysis [20]. AS was defined as an individual whose baPWV value was higher than the study’s 75th percentile (baPWV > 1781 cm/s) [4, 21, 22].

### Statistical analysis

To characterize the distribution of participants’ characteristics, the study population was divided into two groups according to gender. Continuous variables were expressed as mean ± standard deviation (normal distribution) or median (quartile 1-quartile 3) (skewed distribution) and were compared between males and females using analysis of variance or the Kruskal-Wallis H test. Categorical variables were expressed as numbers (percentages), and differences between males and females were tested using the chi-square test or Fisher’s exact test. The association of VFA, SFA, BMI, and WC with baPWV was examined using Pearson’s correlation analysis. Generalized additive modeling (GAM) was used to investigate the relationships between VFA and SFA with baPWV in both men and women. In multiple linear regression and multivariate logistic regression, we transformed VFA and SFA into natural logarithms [23], and we constructed 4 models: Model 1 for adjusted age and sex; model 2 added SBP, DBP, BMI, smoking, alcohol consumption, and work status to the previous model; model 3 added HbA1c, TC, TG, HDL-C, and LDL-C to the previous model; and model 4 added FPG, PPG, FCP, glucose-lowering medication, antihypertensive medication, lipid-lowering medications, diabetes duration, logSFA (model 4 of logVFA), and logVFA (model 4 of logSFA) to the previous model. Multiple linear regression was used to describe the independent associations between logVFA and logSFA with baPWV. Multivariate logistic regression assessed the independent associations of VFA and SFA quartiles with increased AS in patients with T2D. Furthermore, we conducted interaction tests and subgroup analyses to examine the relationship between VFA and AS. Statistical analyses were performed using R version 4.2.0 and EmpowerStats version 4.0.

## Results

### Participants’ characteristics

A total of 1475 patients with T2D were included in this cross-sectional study for analysis; the mean age of the total population was 52.32 ± 10.96 years, of which 854 (57.90%) were male and 621 (42.10%) were female; 157 were menopausal and 464 were non-menopausal among the females. Table 1 describes the characteristics of the participants by comparing different genders. There were no significant differences in SFA, FPG, PPG, FCP, LDL-C, and use of antihypertensive medication between males and females; compared to females, males had a higher BMI, WC, VFA, DBP, TG, and a higher proportion of 47.95% with smoking, 40.73% with alcohol use, 76.76% with a job, and 14.92% with the use of lipid-lowering medication, whereas age, SBP, TC, HDL-C, duration of diabetes, baPWV, and the proportion of 83.14% with the use of glucose-lowering drugs were lower.

**Table 1:** Baseline characteristics of the participants

	Male (*n* = 854)	Female (*n* = 621)	*P*-value
Age (years)	50.30 ± 10.76	55.09 ± 10.63	< 0.001
BMI (kg/m^2^)	26.21 ± 3.48	24.87 ± 3.48	< 0.001
WC (cm)	93.77 ± 9.63	87.73 ± 9.77	< 0.001
VFA (cm^2^)	96.44 ± 42.57	79.81 ± 32.74	< 0.001
SFA (cm^2^)	176.63 ± 60.49	173.72 ± 62.73	0.37
SBP (mmHg)	133.54 ± 18.07	139.39 ± 20.83	< 0.001
DBP (mmHg)	84.91 ± 11.09	81.62 ± 11.43	< 0.001
FPG (mmol/l)	8.87 ± 3.51	8.59 ± 3.22	0.119
PPG (mmol/l)	13.71 ± 5.39	13.29 ± 4.95	0.133
FCP (nmol/L)	0.42 (0.27–0.60)	0.41 (0.28–0.61)	0.908
HbA1c (%)	8.46 ± 2.27	8.09 ± 2.07	0.001
TC (mmol/l)	4.86 ± 1.34	5.06 ± 1.11	0.002
TG (mmol/l)	3.07 (1.33–3.21)	2.32 (1.25–2.60)	< 0.001
HDL-C (mmol/l)	1.17 ± 0.31	1.37 ± 0.32	< 0.001
LDL-C (mmol/l)	2.77 ± 0.92	2.82 ± 0.92	0.256
Duration of diabetes (month)	46.00 (13.00–98.00)	60.00(22.00-111.00)	0.01
baPWV	1587.49 ± 314.13	1646.86 ± 340.12	< 0.001
Smoking (%)			< 0.001
Never	327 (38.34%)	599 (96.46%)	
Former	117 (13.72%)	5 (0.81%)	
Current	409 (47.95%)	17 (2.74%)	
Alcohol consumption (%)			< 0.001
Never	419 (49.18%)	583 (93.88%)	
Former	86 (10.09%)	15 (2.42%)	
Current	347 (40.73%)	23 (3.70%)	
Work (%)			< 0.001
No	198 (23.24%)	434 (70.23%)	
Yes	654 (76.76%)	184 (29.77%)	
Glucose-lowering drugs			0.044
No	144 (16.86%)	81 (13.04%)	
Yes	710 (83.14%)	540 (86.96%)	
Antihypertensive drugs			0.412
No	625 (73.27%)	443 (71.34%)	
Yes	228 (26.73%)	178 (28.66%)	
Lipid-lowering drugs			0.035
No	724 (85.08%)	550 (88.85%)	
Yes	127 (14.92%)	69 (11.15%)	
High baPWV			< 0.001
No	674 (78.92%)	435 (70.05%)	
Yes	180 (21.08%)	186 (29.95%)	

Abbreviations: BMI, body mass index; WC, waist circumference; VFA, visceral fat area; SFA, subcutaneous fat area; SBP, systolic blood pressure; DBP, diastolic blood pressure; FPG, fasting plasma glucose; PPG, postprandial plasma glucose; FCP, fasting C peptide; HbA1c, glycated hemoglobin; TC, total cholesterol; TG, triglycerides; HDL-C, high-density lipoprotein-cholesterol; LDL-C, low-density lipoprotein-cholesterol; baPWV, brachial-ankle pulse wave velocity;

### Correlation of logVFA and logSFA with baPWV

**Fig. 1:**
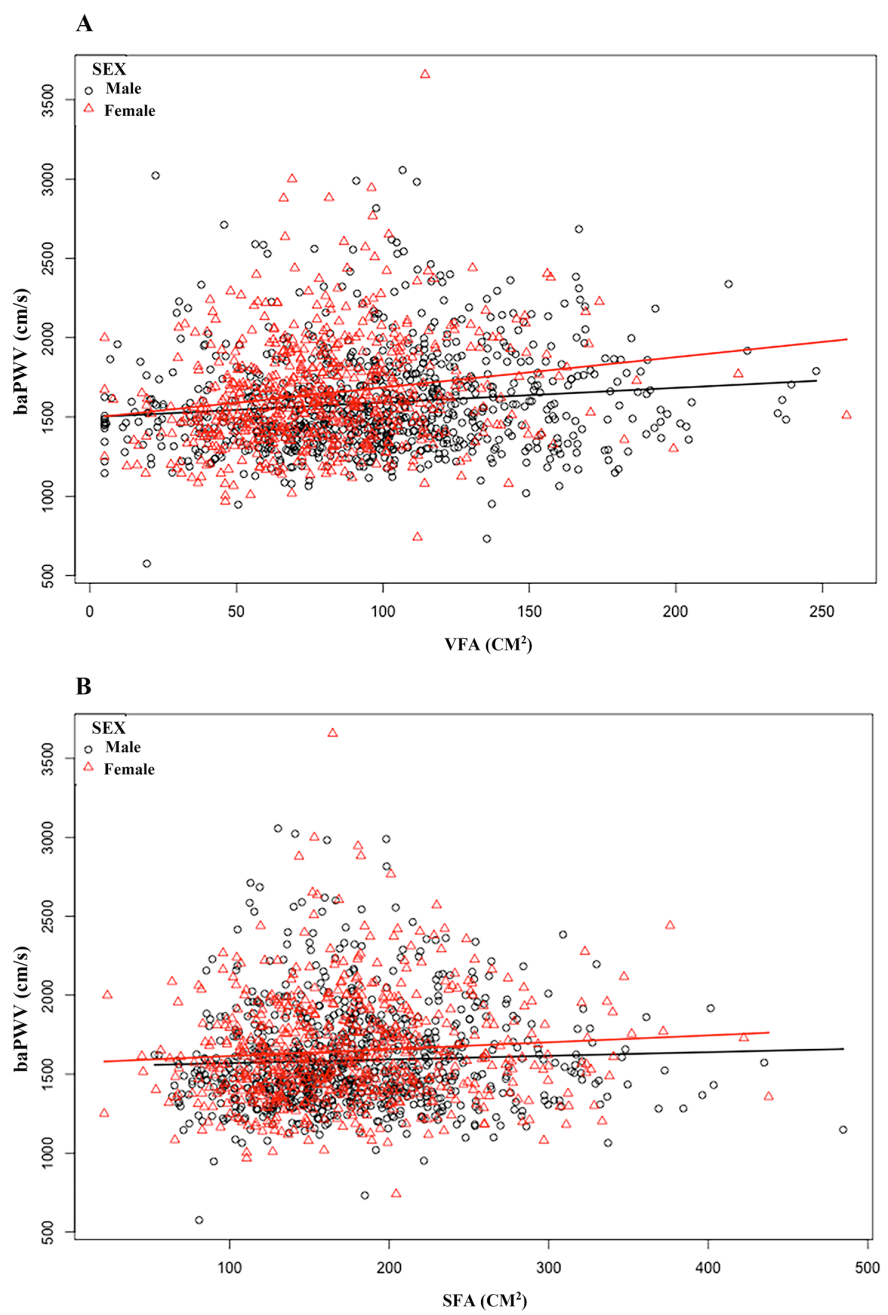
Scatter plots illustrate the relationship between baPWV and body fat parameters in males and females

**Fig. 2:**
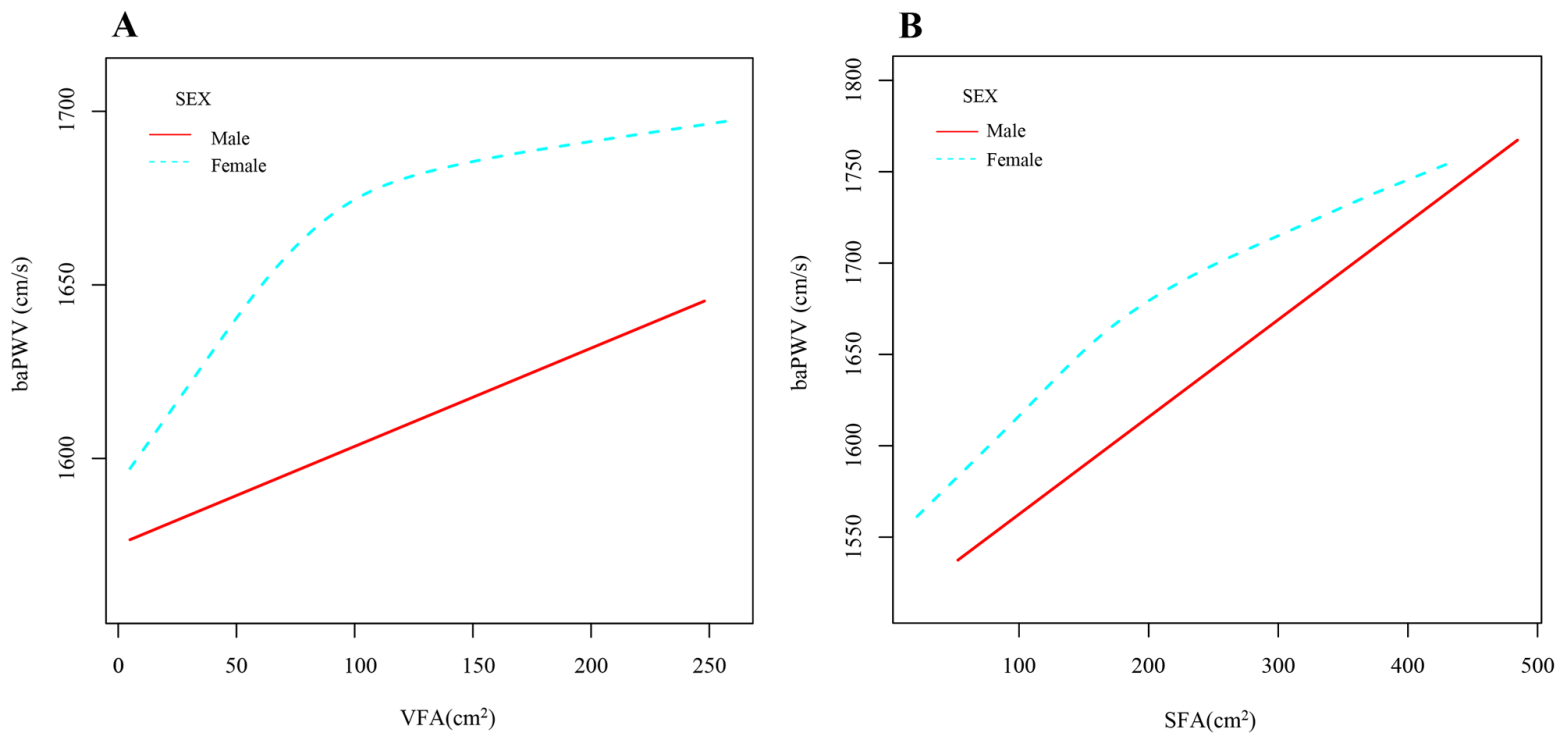
Generalized additive modeling of the relationship between VFA (**A**) and SFA (**B**) with baPWV across sexes after adjustment for age, SBP, DBP, BMI, smoking, alcohol consumption, work status, HbA1c, TC, TG, HDL-C, LDL-C, FPG, PPG, FCP, glucose-lowering medication, antihypertensive medication, lipid-lowering medications, diabetes duration, SFA (in figure B), and VFA (in figure A)

In the Pearson’s correlation analysis in Table 2, baPWV was linked to VFA, WC, and women’s SFA, but not with BMI (*P* = 0.3783) or men’s SFA (*P* = 0.1899). In Fig. 1, these relationships are displayed as scatter plots. We analyzed the correlation of VFA and SFA with baPWV by GAM, and after adjusting for all confounders (Fig. 2A with the addition of the confounder SFA and Fig. 2B with the addition of the confounder VFA), we found that VFA (Fig. 2A) and SFA (Fig. 2B) were positively correlated with baPWV in both men and women. We found in Table 3 by logistic regression modeling that for each 1-unit increase in logVFA and logSFA in model 4 fully adjusted for confounders, the beta coefficient (β) of baPWV increased by 63.1 cm/s (95% confidence interval (CI): 18.4, 107.8) (*P* < 0.05) and 82.4 cm/s (95% CI: -22.8, 187.5) (*P* = 0.125). The logVFA and logSFA quartiles were further grouped and placed as categorical variables in a logistic regression model, with the first group serving as the reference group; in Model 4, the beta coefficients of baPWV were increased by 103.0 cm/s (95% CI: 29.1, 176.9) (P for trend = 0.005) and 79.6 cm/s (95% CI: -2.7, 162.0) (P for trend = 0.195).

**Table 2:** Correlation between baPWV and obesity indicators

Parameter	Total		Male		Female	
	*r*	*P*	*r*	*P*	*r*	*P*
VFA (cm^2^)	0.1234	< 0.0001	0.1256	0.0002	0.1846	< 0.0001
SFA (cm^2^)	0.0588	0.0239	0.0449	0.1899	0.0811	0.0434
BMI (kg/m^2^)	0.0230	0.3783	0.0286	0.4050	0.0555	0.1675
WC (cm)	0.0848	0.0012	0.0861	0.0124	0.1547	0.0001

Abbreviations: VFA, visceral fat area; SFA, subcutaneous fat area; BMI, body mass index; WC, waist circumference;

**Table 3:** Association between logVAF, logSAF with baPWV in different models

	baPWV, cm/s, β (95%CI)			
	Model I *P* value	Model II *P* value	Model III *P* value	Model IV *P* value
logVFA	80.5 (54.4, 106.6) <0.001	53.0 (20.5, 85.5) 0.001	43.6 (9.8, 77.4) 0.012	63.1 (18.4, 107.8) 0.006
logVFA quartile				
Q1	Ref	Ref	Ref	Ref
Q2	44.6 (3.2, 86.0)	20.8 (-18.8, 60.5)	16.9 (-23.5, 57.3)	51.6 (-4.1, 107.2)
Q3	106.7 (65.4, 148.1)	73.9 (30.6, 117.3)	64.1 (19.5, 108.8)	84.3 (23.0, 145.7)
Q4	120.6 (78.6, 162.7)	74.8 (22.0, 127.6)	64.6 (10.7, 118.6)	103.0 (29.1, 176.9)
P for trend	< 0.001	< 0.001	0.005	0.005
logSFA	118.2 (77.2, 159.2) <0.001	129.9 (59.8, 200.1) <0.001	112.9 (41.7, 184.1) 0.002	82.4 (-22.8, 187.5) 0.125
logSFA quartile				
Q1	Ref	Ref	Ref	Ref
Q2	77.5 (36.1, 119.0)	76.1 (35.6, 116.6)	67.6 (26.5, 108.7)	69.9 (13.8, 126.1)
Q3	91.4 (50.0, 132.8)	79.6 (33.0, 126.2)	67.2 (19.8, 114.5)	27.1 (-36.5, 90.7)
Q4	114.9 (73.4, 156.5)	98.5 (37.4, 159.5)	89.6 (28.0, 151.3)	79.6 (-2.7, 162.0)
P for trend	< 0.001	0.002	0.007	0.195

*Notes*: Model I: adjust for age and sex. Model II: adjusts for age, sex, SBP, DBP, BMI, smoking, alcohol consumption, and work status. Model III: adjust for age, sex, SBP, DBP, BMI, smoking, alcohol consumption, work status, HbA1c, TC, TG, HDL-C, and LDL-C. Model IV: adjusts for age, sex, SBP, DBP, BMI, smoking, alcohol consumption, work status, HbA1c, TC, TG, HDL-C, LDL-C, FPG, PPG, FCP, glucose-lowering medication, antihypertensive medication, lipid-lowering medications, diabetes duration, logSFA (model 4 of logVFA), and logVFA (model 4 of logSFA)

Abbreviations: logVFA, the natural logarithm of the visceral fat area; logSFA, the natural logarithm of the subcutaneous fat area; baPWV, brachial-ankle pulse wave velocity; β, beta coefficient; CI, confidence interval;

### Correlation of logVFA and logSFA with increased AS

As shown in Table 4, the risk of AS increased by 50% for each 1-unit increase in logVFA in model 4, fully adjusted by multivariable logistic regression, with an odds ratio (OR) of 1.8 (95% CI: 1.1, 3.1) for elevated baPWV (*P* = 0.019). The logVFA quartiles were further grouped, with the first group serving as the reference group. Results showed that in model 4, adjusted for all confounders, the OR (95% CI) for elevated baPWV was 2.2 (1.2, 3.9), 1.8 (0.9, 3.5), and 2.6 (1.2, 5.5) for groups 2, 3, and 4, respectively, compared with the reference group. The risk of increased AS was statistically significant (P for trend = 0.042). However, we found an OR of 2.4 (95% CI: 0.9, 6.9) for each 1-unit increase in logSFA for elevated baPWV, a result that was not statistically significant (*P* = 0.091). Furthermore, no significant correlation was found between logSFA and the risk of increased AS in model 2 (P for trend = 0.146), model 3 (P for trend = 0.242), or the fully adjusted model 4 (P for trend = 0.299), according to the results of the logSFA quartile groupings.

**Table 4:** Association between logVFA and logSFA with elevated baPWV in different models

	Elevated baPWV > 1,781 cm/sec, OR (95%CI)				
	Model I *P* value	Model II *P* value		Model III *P* value	Model IV *P* value
logVFA	1.9 (1.4, 2.5) <0.001	1.6 (1.1, 2.4) 0.011		1.5 (1.0, 2.3) 0.030	1.8 (1.1, 3.1) 0.019
logVFA quartile					
Q1	Ref	Ref		Ref	Ref
Q2	1.5 (1.0, 2.2)	1.3 (0.9, 2.0)		1.3 (0.8, 2.0)	2.2 (1.2, 3.9)
Q3	2.0 (1.3, 2.9)	1.6 (1.0, 2.6)		1.5 (0.9, 2.5)	1.8 (0.9, 3.5)
Q4	2.6 (1.7, 3.8)	2.0 (1.2, 3.5)		1.9 (1.1, 3.3)	2.6 (1.2, 5.5)
P for trend	< 0.001	0.009		0.026	0.042
logSFA	2.2 (1.5, 3.2) <0.001	2.2 (1.0, 4.6) 0.045		1.9 (0.9, 4.1) 0.102	2.4 (0.9, 6.9) 0.091
logSFA quartile					
Q1	Ref	Ref		Ref	Ref
Q2	1.5 (1.0, 2.2)	1.5 (1.0, 2.3)		1.4 (0.9, 2.1)	1.5 (0.9, 2.7)
Q3	1.7 (1.2, 2.5)	1.5 (0.9, 2.5)		1.4 (0.8, 2.3)	1.3 (0.7, 2.5)
Q4	2.0 (1.4, 3.0)	1.6 (0.9, 3.1)		1.5 (0.8, 2.9)	1.7 (0.8, 4.0)
P for trend	< 0.001	0.146		0.242	0.299

*Notes*: Model I: adjust for age and sex. Model II: adjusts for age, sex, SBP, DBP, BMI, smoking, alcohol consumption, and work status. Model III: adjust for age, sex, SBP, DBP, BMI, smoking, alcohol consumption, work status, HbA1c, TC, TG, HDL-C, and LDL-C. Model IV: adjusts for age, sex, SBP, DBP, BMI, smoking, alcohol consumption, work status, HbA1c, TC, TG, HDL-C, LDL-C, FPG, PPG, FCP, glucose-lowering medication, antihypertensive medication, lipid-lowering medications, diabetes duration, logSFA (model 4 of logVFA), and logVFA (model 4 of logSFA)

Abbreviations: logVFA, the natural logarithm of the visceral fat area; logSFA, the natural logarithm of the subcutaneous fat area; baPWV, brachial-ankle pulse wave velocity; OR, odd ratio; CI, confidence interval;

### Subgroup analysis of logVFA and increased risk of AS

**Fig. 3:**
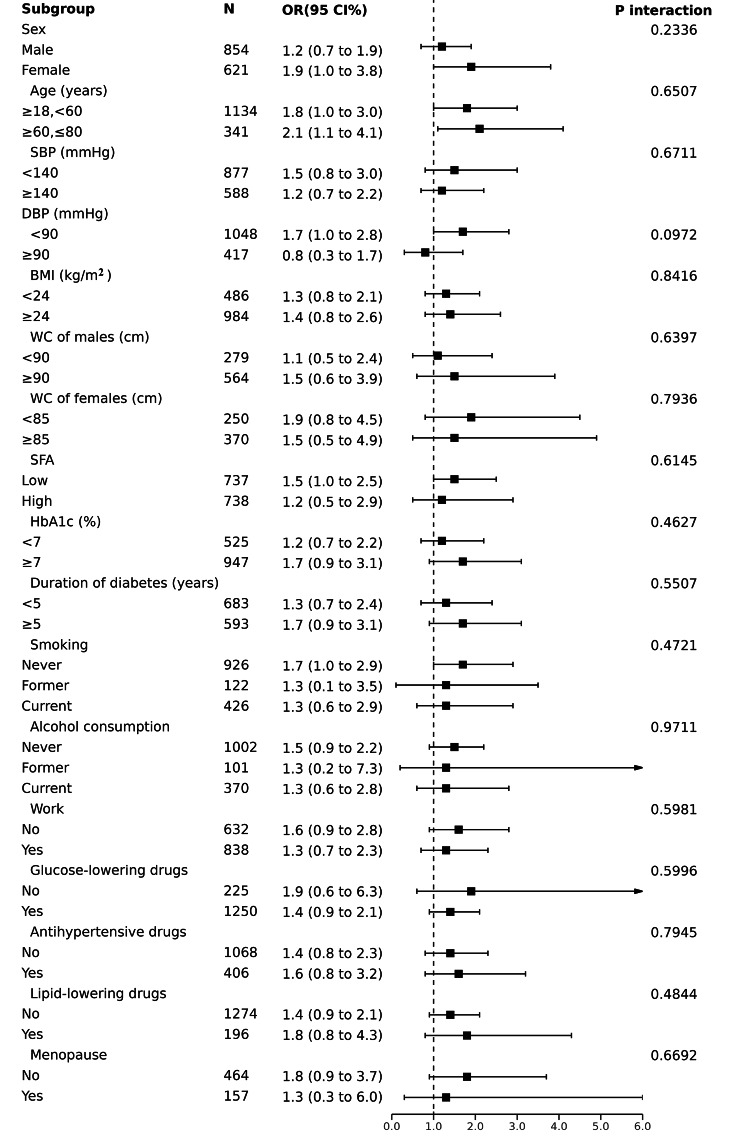
Subgroup analyses for the association between logVFA and elevated baPWV were adjusted for age, sex, SBP, DBP, BMI, smoking, alcohol consumption, work status, HbA1c, TC, TG, HDL-C, LDL-C, FPG, PPG, FCP, glucose-lowering medication, antihypertensive medication, lipid-lowering medications, diabetes duration, and logSFA, except for the stratified variable

To further explore the correlation between logVFA and increased AS in different populations, we performed subgroup analyses (Fig. 3). logVFA was positively associated with the risk of increased AS in all subgroups except the DBP ≥ 90 mmHg group. We dichotomized the SFA groups, and subgroup analyses showed a positive correlation between logVFA and AS in both the low and high SFA groups. After excluding the stratification factor itself and adjusting for effect modifiers, there was no interaction between subgroups (P interaction > 0.05), and the results were stable. Although logVFA was negatively associated with an increased risk of AS at DBP ≥ 90 mmHg and positively associated at DBP < 90 mmHg, there was no interaction in this subgroup (P interaction = 0.0972).

## Discussion

In this study, the correlation between VFA and SFA with AS in patients with T2D was assessed by baPWV. This cross-sectional study showed a significant positive and dose-response relationship between VFA and baPWV in both males and females, while the dose-response relationship between SFA and baPWV was not significant in males. Furthermore, in multiple logistic regression model 1, We found that VFA and SFA were significantly positively correlated with AS when adjusting only for age and sex, but after adjusting for all confounders, the positive correlation between VFA and AS remained significant; however, the positive correlation between SFA and AS was not significant. Our study confirms a stronger, independent positive association between VFA and an increased risk of AS compared to SFA.

Abdominal fat consists of internal VFA and SFA, and abdominal obesity has a stronger correlation with AS than does total obesity [10]. However, most of the previous studies have only discussed the correlation between VFA and AS, and only a smaller number of studies have compared the correlation between VFA and SFA with AS. The sample sizes of these studies were small, and in addition, there is currently controversy regarding the correlation between SFA and AS [5–8].

The study by Ryotaro et al. included 148 T2D patients (mean age 65 ± 12 years) in China. The study showed that high VFA and low SFA are determinants of AS in patients with T2D, and high SFA protects against AS in patients with T2D. However, the study used carotid intima-media thickness (CIMT) to assess AS [7]. This is different from our results. This may be related to the different methods used in this study-like assessment of AS. Alterations in the intimal, middle, and tunica layers of the vasculature, both structurally and functionally, are linked to AS. It is controlled by elements coming from various levels of the vessel wall as well as chemicals like insulin and aldosterone in plasma [24]. Studies have shown that CIMT is mainly used as a measure of localized vascular lesions, whereas baPWV is currently used as a measure of AS [25–27]. A study by Amparo et al. that included 415 European Americans with an average age of 55 years old confirmed that VFA was independently associated with increased AS and was an independent predictor of CVD, while SFA and BMI did not predict CVD [5]. Another study by Melanie et al. involved 44 early T2D patients between the ages of 30 and 70. They found that VFA was an independent risk factor for arterial inflammation (a marker of early AS), whereas SFA was not. In addition, WC was not associated with arterial inflammation. The study demonstrated that, in comparison to SFA, VFA was more strongly related to early AS in T2D patients [6]. This is consistent with our findings. Studies have shown that both BMI, which assesses overall obesity, and WC, which assesses central obesity, do not correlate as strongly with AS as VFA [12, 13], and a large number of studies have reported no significant correlation between BMI and WC and AS [5, 6, 10, 14]. Furthermore, numerous previous studies have shown that the visceral adiposity index (VAI), which responds to visceral fat, is independently and positively correlated with AS [28–30]. In a study involving 5921 Persian adults, Zahra et al. found that among overweight men and women, as well as obese women, VFA linked better with AS than VAI, BMI, and WC [31]. The VAI, which includes lipid-related metabolic markers (TG and HDL-C) and anthropometric indices (BMI and WC), is a newly developed visceral fat index that is thought to be able to depict the distribution of fat [28], but the VFA predicts AS risk better than it. Visceral fat accumulation is more common in men and menopausal women, while subcutaneous fat accumulation is more common in women who are not menopausal [32, 33]. Through the use of general linear correlation analysis, we discovered that baPWV had a stronger association with VFA than with WC, but not with BMI. In women, baPWV is correlated with SFA, but not in men. Because of the different fat distribution in men and women, we found that VFA was significantly and positively linked with AS in both men and women by subgroup analysis. Furthermore, we performed subgroup analyses of whether women were menopausal or not, taking into account changes in hormone levels and altered lipid metabolism in women before and after menopause due to changes in hormone levels in the body [34], and it was discovered that in both menopausal and non-menopausal women, VFA was positively correlated with AS.

Our study found a stronger correlation between VFA than SFA and AS, probably because visceral adipose tissue (VAT) is composed of larger, insulin-resistant adipocytes, which are mainly distributed in the abdominal cavity organs such as the liver and heart [35]. Research has indicated that an increase in VAT also significantly increases the portal vein’s ability to transfer free fatty acids into the liver, which can lead to hepatic IR and elevated LDL-C production and release [8]. Subcutaneous adipose tissue (SAT), which is mainly distributed between the dermis and the fascia, secretes more leptin and lipocalin than VAT and promotes glucose and lipid metabolism [36]. Insulin sensitivity and glucose tolerance were observed to improve in animal trials when SAT was transplanted into the visceral cavity [37]. VAT produces pro-inflammatory cytokines, recruiting more pro-inflammatory macrophages and secreting more vasoconstrictive mediators than SAT [4, 38, 39]. Studies have shown that in people with the same BMI, the risk of CVD increases with the accumulation of visceral fat [40]. In our subgroup analysis, we found that using the Chinese diagnostic criteria for obesity, when the BMI used to diagnose overall obesity was normal (< 24 kg/m^2^) and when the WC used to diagnose central obesity was normal (men < 90 cm and women < 85 cm), VFA remained positively linked to AS after all confounders were excluded. Although the occurrence of CVD events is more prominent in obese T2D patients, this study found that VFA is an independent risk factor for CVD in T2D patients with normal weight. Therefore, physicians should pay attention to the monitoring of VFA in T2D patients when treating diabetes mellitus to reduce the occurrence of CVD events.

Our study does, however, have certain limitations. Firstly, because it is cross-sectional in nature, it is not possible to draw conclusions about the causal relationship between VFA and SFA with baPWV. Secondly, this study used BIA to measure VFA and SFA. A more objective assessment of the buildup of abdominal fat necessitates abdominal computed tomography or magnetic resonance imaging measurements. However, accumulating evidence has confirmed that the BIA is more cost-effective, simple to perform, and non-invasive in estimating VFA and SFA in diabetic patients [41–44]. Thirdly, recall bias cannot be completely ruled out due to this study design, but future follow-up data from MMC may provide more precise evidence. Finally, this study only included Chinese patients with T2D; more research is required to determine whether the findings apply to people of other races.

## Conclusions

Our study showed that VFA was significantly positively associated with AS and stronger than SFA in patients with T2D. After adjusting for confounders, VFA remained significantly and independently positively associated with AS, whereas SFA was not significantly associated with AS. Therefore, we further emphasize the importance of monitoring and lowering VFA in patients with T2D to reduce the risk of CVD.

## Data Availability

The corresponding author will provide raw data supporting the conclusions of this paper without reservation upon reasonable request.

## Abbreviations

VFA: Visceral fat area
SFA: Subcutaneous fat area
AS: Arterial stiffness
T2D: Type 2 diabetes
baPWV: Brachial-ankle pulse wave conduction velocity
GAM: Generalized additive model
CI: Confidence interval
CVD: Cardiovascular disease
BIA: Bioelectrical impedance analysis
IR: Insulin resistance
BMI: Body mass index
WC: Waist circumference
MMC: Metabolic Management Center
FPG: Fasting plasma glucose
HbA1c: Glycated hemoglobin
TC: Total cholesterol
TG: Triglycerides
HDL-C: High-density lipoprotein cholesterol
LDL-C: Low-density lipoprotein cholesterol
FCP: Fasting C peptide
PPG: Postprandial plasma glucose
SBP: Systolic blood pressure
DBP: Diastolic blood pressure
β: Beta coefficient
OR: Odds ratio
CIMT: Carotid intima-media thickness
VAT: Visceral adipose tissue
SAT: Subcutaneous adipose tissue
